# Bowls, vases and goblets—the microcrockery of polymer and nanocomposite morphology revealed by two-photon optical tomography

**DOI:** 10.1038/s41467-021-25297-w

**Published:** 2021-08-20

**Authors:** Shu-Gui Yang, Zhen-Zhen Wei, Liliana Cseh, Pantea Kazemi, Xiang-bing Zeng, Hui-Jie Xie, Hina Saba, Goran Ungar

**Affiliations:** 1 State Key Laboratory for Mechanical Behaviour of Materials, Shaanxi International Research Centre for Soft Matter, Xi’an Jiaotong University, Xi’an, China; 2 Department of Materials Science and Engineering, University of Sheffield, Sheffield, UK; 3 College of Textile and Clothing Engineering, National Engineering Laboratory for Modern Silk, Soochow University, Suzhou, China; 4 Romanian Academy, Coriolan Dragulescu Institute of Chemistry, Timisoara, Romania; 5 Department of Physics, Zhejiang Sci-Tech University, Hangzhou, China

**Keywords:** Nanocomposites, Polymers, Composites, Confocal microscopy

## Abstract

On the >1 µm scale the morphology of semicrystalline plastics like polyethylene or Nylon features spherulites, “shish-kebabs”, cylinddrites and other crystalline aggregates which strongly affect mechanical and other material properties. Current imaging techniques give only a 2D picture of these objects. Here we show how they can be visualized in 3D using fluorescent labels and confocal microscopy. As a result, we see spherulites in 3D, both in neat polymers and their nanocomposites, and observe how unevenly nanoparticles and other additives are distributed in the material. Images of i-polypropylene and biodegradable poly(lactic acid) reveal previously unsuspected morphologies such as “vases” and “goblets”, nonspherical “spherulites” and, unexpectedly, “shish-kebabs” grown from quiescent melt. Also surprisingly, in nanocomposite sheets spherulite nucleation is seen to be copied from one surface to another, mediated by crystallization-induced pressure drop and local melt-flow. These first results reveal unfamiliar modes of self-assembly in familiar plastics and open fresh perspectives on polymer microstructure.

## Abstract (web summary)

The morphology of semicrystalline plastics on the 1-100 μm scale, such as spherulites, strongly affect mechanical and other properties of the material but currently only 2D imaging techniques are available. Here, the authors use fluorescence labels and confocal microscopy to visualize the internal structure of neat polymers and composites in 3D and reveal unsuspected morphologies.

## Introduction

Spherulites, shish-kebabs, cylindrites, and other morphological features of bulk semicrystalline polymers (SCP) have so far been studied using methods such as polarized optical microscopy (POM)^1^, transmission electron (TEM)^2^, or atomic force microscopy (AFM)^3^. For these studies either thin films or thin sections were used, giving 2D but not 3D pictures. The organization in the 3rd dimension has been implied rather than observed, and confined to tedious and destructive microtome sectioning. True 3D imaging of SCP morphology has not yet been reported.

Electron tomography has been used for visualizing chemically heterogeneous structures of polymer blends^4^, block copolymers^5–8^ and nanoparticle assemblies in block copolymers^9^ and polypeptides^10^. The method is successful when absorption contrast exists between chemically different species and where the structures are on a ≤1 µm scale^11^. However it is not at its best for studying SCP morphology where (a) the objects (e.g. spherulites) are on a scale of tens and hundreds of µm and (b) where there is no chemical contrast. In addition, exposing a SCP to a very high irradiation dose required by electron tomography will completely destroy the crystallinity of an organic polymer, and with it, the only available contrast, that between the crystalline and amorphous phase.

Recently, more exotic 3D imaging techniques have been tried, such as using X-rays based on the minute difference in X-ray refractive index in polymer blends^12^ and foams^13^. Despite some success, these methods suffer from low contrast and require highly specialized and scarce coherent synchrotron X-ray beamlines.

Regarding SCP nanocomposites, the distribution of nanoparticles (NPs) in these systems is still relatively poorly understood. TEM of thin sections had some success in locating metal NPs but giving a very localized picture^14^. The location of silica NPs in polymers like polypropylene and poly(ethylene oxide) has been studied because of the commercial importance of such nanocomposites. However, due to difficulties visualizing submicron SiO_2_ NPs only diffraction techniques were used, such as small-angle X-ray or neutron scattering (SAXS, SANS), giving a spatially averaged picture of NP aggregation rather than an image of their location^15^. In fact even 2D imaging has been missing. For example, it had been assumed that as spherulite grows during polymer solidification, small NPs (<5 nm) could be excluded but larger ones would stay trapped within the spherulites, thus remaining as uniformly dispersed in the solid polymer as in the precursor melt. However, our preliminary 2D studies using labeled NPs have shown that, contrary to theoretical predictions, large NPs are to a considerable extent pushed ahead of growing spherulites and deposited at their boundaries^16^ with potentially serious consequences for material integrity.

**Fig. 1:**
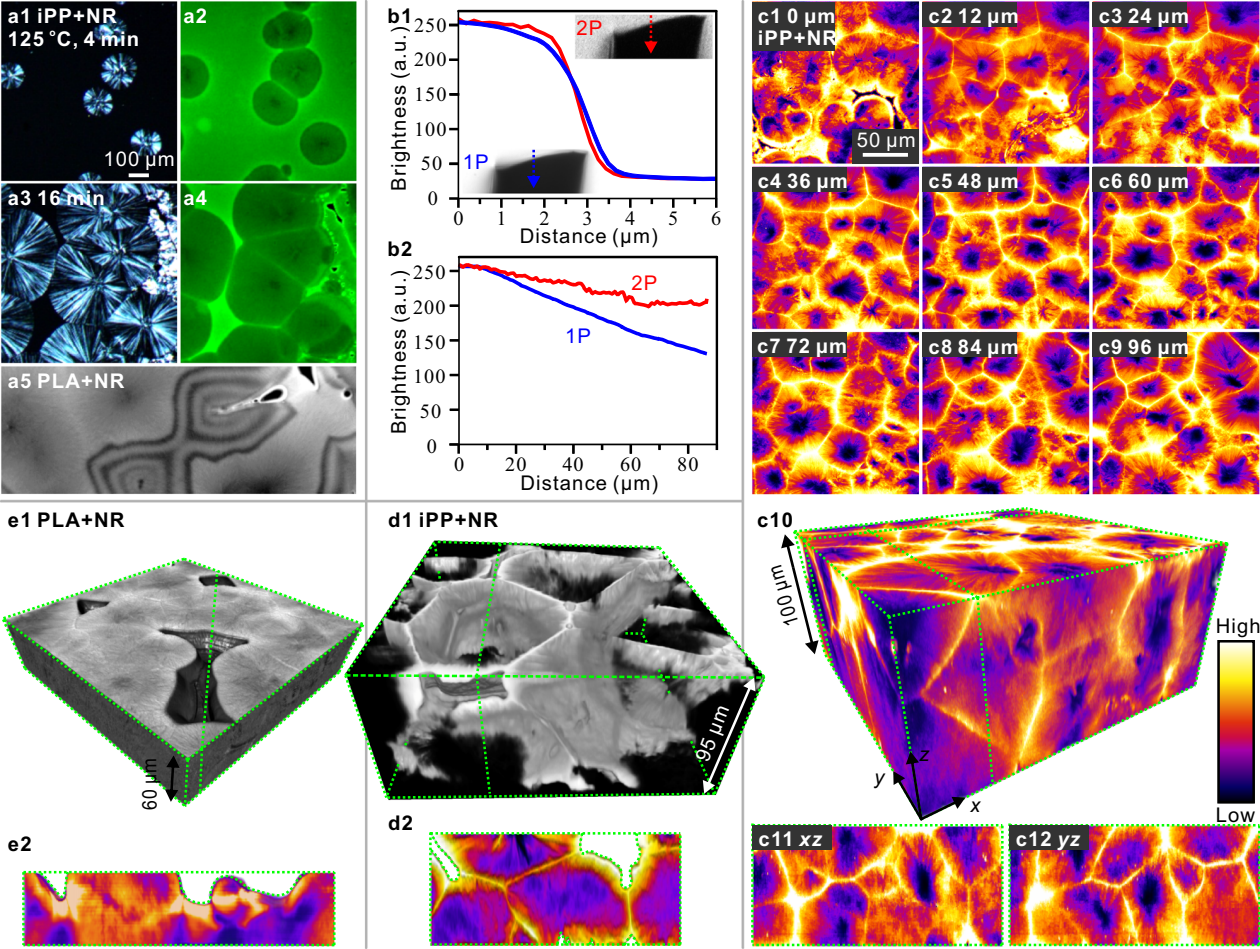
2D and 3D fluorescence microscopy of fluorophore-labeled polymers after complete isothermal crystallization showing internal and surface structure. **a1**-**a4**, 2D micrographs of ∼10 µm thick film of iPP doped with 0.05 wt % Nile Red during isothermal crystallization at 125 °C: (**a1**, **a3**) cross-polarized and (**a2**, **a4**) FM; the dye is seen to be partially rejected from the growing spherulites. **a5** FM of fully crystallized PLA; cavitation and Newton interference contours are seen. **b** Comparison of 1-photon and 2-photon confocal microscopies: (b1) *z-*scans across a sharp end of a glass fiber embedded in NR-doped polystyrene (PS) — blue = 1-photon, red = 2-photon; (b2) 1-photon (blue), and 2-photon (red) fluorescence intensity *vs*. depth in a sheet of NR-doped PS illuminated by 543 nm and 1000 nm lasers, respectively. **c** NR-labeled iPP crystallized at 120 °C: (**c1**–**c9**) selected *z-*slices as recorded by 2-photon microscopy, bottom to top, using false-color scale at the bottom right; (c10) 3D solid reconstructed therefrom (see Supplementary Video 1); (c11,c12) vertical (*xz* and *yz*) slices along dashed lines in c10. **d** Labeling by infiltration: neat iPP was crystallized at 130 °C and subsequently immersed in NR/p-xylene solution; (d1) “porous” 3D rendering; (d2) vertical section along the dotted line in **d1**. **e** NR-doped PLA crystallized at 120 °C showing surface cracks (*cf*. 2D image in **a5**); (**e1**) gray scale solid (bright = high fluorescence, black = 0); (**e2**) vertical slice along dashed line in (**e1**). Exceptionally, the crack areas (zero fluorescence) in (**d2**) and (**e2**) are shown white.

Beside spherulites, cylindrites, “shish-kebabs,” and transcrystalline layers^17–19^, interest in morphology on the µm to mm scale in SCPs also includes cavities produced during solidification^20^ or cooling. Polymer spherulites are aggregates of thin (10–50 nm) crystalline lamellae grown mainly radially from the common nucleus^21–23^. The classic method of viewing them is POM. In Fig. 1(a1, a3) the birefringent spherulites of isotactic polypropylene (iPP) are seen to grow from the isotropic melt. However this method is inevitably restricted to thin films, normally significantly thinner than the spherulite diameter, thus imaging disks rather than spheres. The long-standing question is how to visualize spherulites and, generally, polymer morphology on this scale in 3D.

## Results and discussion

An appropriate contrast method is required, as a start. We were inspired by the work of Calvert et al.^24–26^ who used fluorescence microscopy (FM) to view the distribution of a UV absorber in iPP and observed a lowered concentration of the additive within the spherulites. In Fig. 1(a2, a4) we show FM images recorded during the same crystallization run and from the same area as in the POM images in Fig. 1(a1, a3). The iPP had the hydrophobic dye Nile Red (NR) added, initially uniformly dispersed (Supplementary Fig. S1 in Supplementary Information). The low-fluorescence circles are seen to coincide with the birefringent “spherulites,” i.e., disks, in POM images. Moreover, the melt close to the growing spherulites is visibly brighter, as the dye is partially rejected and concentrated ahead of the spherulite growth front. A study by 2D FM of spherulite growth in NR-doped iPP and poly(lactic acid) (PLA) is reported in ref. ^16^.

Another advantage of FM is its ability to clearly image cracks and cavities that are mostly overlooked by POM^27^—see Fig. 1(a5). This fluorescence micrograph, taken from a fully crystallized film of NR-doped PLA, shows a cavity (black, top right) surrounded by a dye-rich polymer. It also shows Newton fringes outlining the areas where the polymer had detached from the glass due to its contraction upon crystallization. The effect of crystallization-induced negative pressure^28^ will help explain some of the observations described further on.

### The technique

In order to use fluorescence to observe the morphology in 3D, we employ confocal laser microscopy. In materials science it has been utilized in studies of polymer blends and block copolymers^29–31^, relying on the difference in solubility or binding of the dye in the chemically different domains. We are aware of only one, indeed a very interesting recent use of this technique in a thin-film homopolymer, where a mechanically cleavable group was incorporated into the chains to give fluorescent radicals, thus marking stress points in the crystallizing material^32^. To observe true 3D morphology in bulk homopolymers here we create contrast in two different ways: (a) by first producing a uniform blend of polymer and fluorophore and then allowing partial exclusion of the fluorophore from the growing spherulites, which results in the dye being concentrated at spherulite boundaries; (b) by immersing the already crystallized polymer in a solution of a fluorescent dye and relying on the dye’s preferential diffusion and deposition along spherulite boundaries. The fluorophore for both methods (a and b) used in this work for imaging pure polymers was commercial Nile Red. For the study of nanocomposites it was the silica nanoparticles themselves that acted as the labels, 200 nm in diameter, highly monodisperse and chemically modified with a custom-synthesized NR derivative—see Supplementary Fig. S2^16^.

**Fig. 2:**
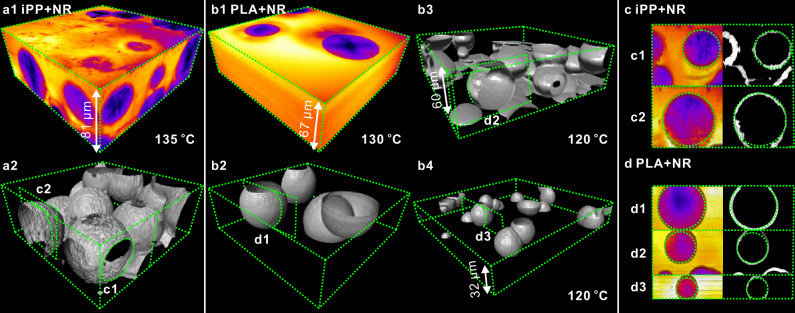
3D images of growing spherulites in NR-labeled polymers. **a** iPP isothermally crystallized at *T*_*c*_ = 135 °C for 12 min, then quenched in ice water, showing spherulites whose growth had been arrested: (**a1**) false-color solid representation (color scale as in Fig.1); (**a2**) same region, showing surfaces of a maximum gradient in fluorescence intensity, thus marking the envelope of arrested spherulites. **b** The same for PLA: (**b1**, **b2**) false-color solid and maximum gradient (surface) rendering, respectively, of the same region, *T*_*c*_ = 130 °C; (**b3**, **b4**) two PLA samples crystallized at *T*_*c*_ = 120 °C. **c**, **d** Vertical sections through selected spherulites of (**c**) iPP in panel **a**, and **d** PLA in panel **b**; left: false color, right: maximum gradient rendering. Sections shown in **c**, **d** are indicated in the corresponding panels **a**, **b**, respectively. Note some vertical elongation of certain spherulites, especially near sample surface, made clear by comparison with the inscribed green reference circles. Note also the difference between the “wrinkly” surface of iPP and the smooth surface of PLA spherulites.

**Fig. 3:**
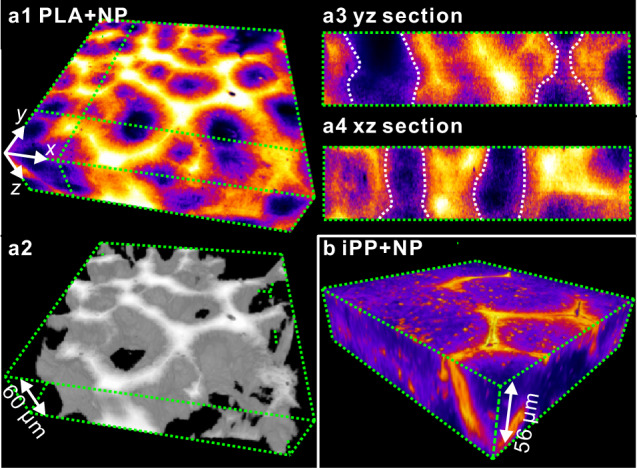
3D morphology of fully crystallized polymers with NR-labeled silica nanoparticles. **a** PLA containing 1.3 wt % of 200 nm NR-labeled nanoparticles fully crystallized at 120 °C. **a1** False-color solid (color scale as in Fig. 1); (**a2**) “porous” rendering of the same region; (**a3**, **a4**) vertical *yz* and *xz* sections along the green dotted lines in **a1**. Note some preference of spherulites to form vertical columns. **b** iPP containing 1.3 wt % of NR-labeled 200 nm SiO_2_ NPs fully crystallized at 135 °C. Both in PLA and in iPP the NPs tend to concentrate at spherulite boundaries.

To create a 3D image, the sample is *xy*-scanned by exciting laser layer by layer and subsequently the slices are combined in a *z-*stack and visualized in a variety of ways, as shown in Figs. 1–3. We used the 2-photon fluorescence method, whereby a near-IR laser is employed for scanning, causing upconversion and fluorescence emission of visible light^33^. The 2-photon method has several advantages over the more conventional 1-photon method^34,35^. First, the resolution in *z-*direction is higher, as illustrated in Fig. 1(b1). In simple terms, this can be understood by assuming that fluorescence intensity scales with the square of exciting light intensity, in the same way as the rate of a second-order chemical reaction A + A → A_2_ is proportional to [A]^2^. Accordingly, for a Gaussian *z-*profile of exciting light intensity, we have also a Gaussian of fluorescence but with a smaller halfwidth, $\sqrt{2}/2$, of that of the exciting light. In principle, the same should apply to *xy* resolution but since this is higher than *z-*resolution anyway and determined by the diffraction limit, the above advantage of 2-photon excitation is canceled by its longer wavelength. Limiting the fluorescence to a smaller excitation volume also has the effect of increasing the signal-to-noise ratio. A further advantage is the deeper penetration of IR light compared to visible, due to its weaker scattering on the optically inhomogeneous spherulitic polymer—see Fig. 1(b2) where attenuation at increasing depth in polystyrene is compared for 1- and 2-photon fluorescence of NR. It is worth recalling that IR photography is used for a clearer view in misty conditions for the same reason. A yet further advantage is reduced photo-bleaching by the lower energy laser.

### Pure dye-labeled polymers

In this work, an *xy-*scan was recorded in 1 µm *z-*increments. As an example, Fig. 1(c1-c9) shows nine slices, 12 µm apart, from an NR-doped iPP sample using false colors to represent fluorescence intensity (see the scale at the bottom right). The 3D image constructed from the full *z-*stack is shown as a solid slab in Fig. 1(c10) and an *xz* and a *yz* slice in Fig. 1(c11) and (c12), respectively—see also Supplementary Video 1. The boundaries between spherulites can be clearly seen as straight bright lines in either *xy*, *xz*, or *yz* planes. This is a result of the dye having been partially rejected from the growing spherulites and remaining concentrated at boundary planes after the spherulites had collided. We note, furthermore, that the dye concentration inside the spherulites is not uniform; it is the lowest in the center (black). This has already been noted previously in 2D fluorescence micrographs^16,25^ and was tentatively attributed to a higher crystallinity of spherulite centers. However, the higher fluorescence away from the center is also partially due to the back diffusion of the dye from the boundaries after crystallization^16^.

If the sole aim is to visualize the morphology rather than investigate the distribution of additives, the fluorescent label can be introduced after sample solidification. This is illustrated in Fig. 1d where NR had been infiltrated into solid iPP by sample immersion in a solution of the dye. A suitable immersion time has to be used; examples of under- and over-immersion are shown in Supplementary Fig. S3. The dye can be seen again to occupy spherulite boundaries, this time because they present preferred diffusion pathways and increased swelling sites. Figure 1(d1) also illustrates another method of 3D visualization, referred to here as “porous” rendering, whereby only fluorescence above a certain threshold is registered; this leaves spherulite interior transparent—see also Supplementary Video 2. Porous rendering can be applied irrespective of whether the dye-infiltration or dye-rejection method is applied, or whether pure polymers or their composites are imaged (see also Fig. 3(a2)).

The images show directly how after crystallization the polymer volume is divided into tessellating irregular Voronoi polyhedra that are conventionally referred to as spherulites, and that are the topological duals of the nucleation centers.

Beside showing the internal structure, 3D confocal imaging is useful for mapping surface irregularities, which can, moreover, be accurately related to the underlying internal morphology, such as spherulite boundaries. Figure 1(d1, d2) of NR-labeled iPP again provides a good example, where the vertical slice in (d2) is taken along the dotted line in (d1). The cracks (white in (d2)) are seen to run along spherulite boundaries, appearing as double walls between spherulites. Propagation of internal cracks can be clearly followed in their entirety by manipulating the 3D-rendered landscape, as seen in the video. Another example of visualization of surface irregularities and internal cavities is presented in Fig. 1e for PLA.

In order to observe 3D morphology during the growth of spherulites before they collide, and test if they are really spherical, their isothermal growth was interrupted by quenching. Selected 3D images of arrested spherulites of iPP are shown in Fig. 2a, c, and of PLA in Fig. 2b, d. False-color solids in Fig. 2(a1, b1) show the low-fluorescent (blue) spherulites surrounded by highly fluorescent (yellow) quenched melt. In iPP the brighter halo surrounding some spherulites can also be seen, coming from the rejected dye. The darker spherulite centers are even more evident than in Fig. 1, as the back-diffusing dye has not had a chance to reach them^16^.

A particularly useful rendering technique for part-crystallized polymers is to highlight interfaces with the maximum gradient in fluorescence intensity. This outlines the spherulite surface very clearly and is applied in Fig. 2(a2, b2–4) and Supplementary Videos 3 and 4. We are not aware of any previous in-situ images of spherical spherulites. But how spherical are they? That the discs commonly observed in *xy* plane by POM are circular is well established. What about the third dimension? Fig. 2(c, d) show vertical sections through selected spherulites from Fig. 2(a, b). Comparing their vertical section with the inscribed reference circles, we can say that the spherulites are closely spherical but elongate somewhat when approaching the polymer-glass interface—more on this in the next section.

### Polymers with nanoparticles

We now turn to polymers containing nanoparticles. While the presence of the NPs did not have a significant effect on spherulite growth rate^16^, it increased their nucleation rate ∼fivefold in the PLA + NP blend—see Supplementary Figs. S4 (calorimetry), S6 (POM), and S7 (graph). Figure 3(a1–a4) show part of a fully crystallized sheet of PLA containing 200 nm dye-labeled silica NPs. Similar morphology is also seen in an equivalent nanocomposite of iPP in Fig. 3b. The observed contrast relies entirely on the redistribution during spherulite growth of the NPs originally homogeneously dispersed in the melt. Had they all been immobile and remained occluded within the spherulites, as theory based on Stokes–Einstein law had predicted^36^, images in Fig. 3 and in another subsequent figure that also shows the morphology of NP-containing polymer would have been featureless. In fact, the theory gives the diffusion rate of 200 nm NPs a thousand times lower than needed to escape a growing spherulite^16^. While NP aggregation on polymer matrix crystallization has been observed by SAXS^36^, the location of the aggregates was unknown. A possible explanation of the unsuspected ability of NPs of this size to be “pushed” and excluded from the growing spherulites is the existence of a depletion layer ahead of the growth front, its high negative pressure^20,28^ pulling-in the particles^16^. More unsuspected and profound consequences of such negative pressure are described below in subsequent figures.

**Fig. 4:**
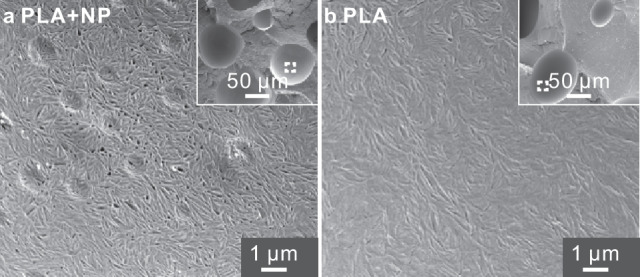
SEM images of PLA fracture surface with and without nanoparticles. **a** PLA with 1.3 wt.% of 200 nm silica NPs—zoom-in of fracture surface inside the spherical hole left by a detached spherulite (marked square in the wider-area inset). Holes left by individual nanoparticles and some of their clusters are seen, as well as numerous gaps between packets of lamellar crystals created by the intercalated NPs. Note that if the NPs were evenly distributed throughout the sample volume, they would have occupied only 0.7 % of the surface. **b** The same for PLA without NPs for comparison; the surface is markedly smoother.

The heightened presence of nanoparticles at the spherulite growth front is also illustrated by SEM of fractured part-crystallized PLA nanocomposite (Fig. 4a). The surface shown is that below a detached spherulite. Unlike the rather smooth surface of a similarly prepared pure PLA sample in Fig. 4b, the boundary surface of the nanocomposite is full of holes and interlamellar gaps left by the numerous accumulated NPs. In comparison, SEM of a fracture surface of amorphous PLA + NP blend of the same composition, quenched from the melt to below *T*_*g*_ and shown in Supplementary Fig. S5, shows only a few isolated single-particle holes.

An interesting feature of Fig. 3a is that in many cases the spherulites tend to appear roughly above each other, forming columns—see Fig. 3(a3, a4) and the Supplementary Video 5 of the “porous” 3d landscape in Fig. 3(a2). This is unexpected, as it is normally assumed that spherulites nucleate randomly and independently.

**Fig. 5:**
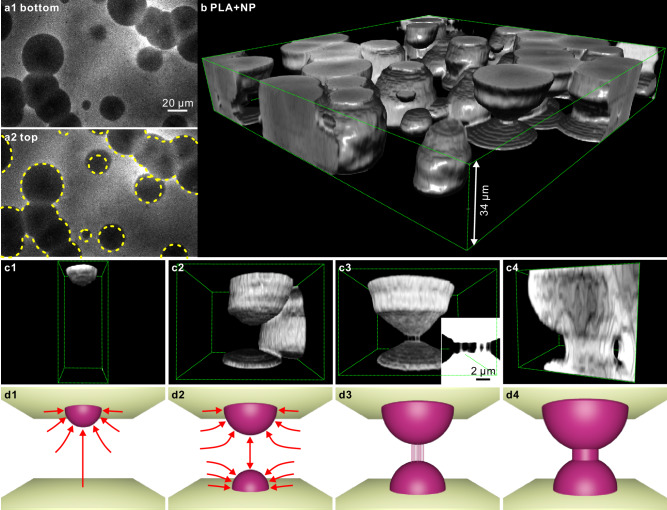
Nucleation mirroring, shish-kebabs from “quiescent” melt and goblet morphology in PLA nanocomposite. PLA containing 1.3 wt% of 200 nm NR-labeled silica NPs was crystallized isothermally at *T*_*c*_ = 130 °C; after 10 min spherulite growth was arrested by quenching in ice water. This applies to all panels in this figure. **a** *z-*slices (**a1**) near the bottom and **a2** near the top surface, illustrating nucleation mirroring across the sheet thickness; the dashed circles in (**a2**) show the exact position of the spherulites directly opposite at the bottom surface seen in (**a1**). **b** Surface rendering of arrested spherulite growth in a 34 µm film; the morphology resembles a treasure of ancient crockery. See also Supplementary Video 6. **c** Selection of images reconstructing different stages of formation of a “goblet”, starting with (**c1**) a single spherulite, (**c2**) formation of a second spherulite at the opposite (bottom) surface, (**c3**) close approach of the two growing spherulites and the formation of bridging shish-kebabs, and finally, (**c4**) formation of the goblet stand by multiplication and radial growth of the shish-kebabs or cylindrites. The inset in (**c3**) is a section through the center of the goblet with inverted contrast (bright = low fluorescence). Panels (**c1**–**c4**) actually show different coexisting regions of the sample. **d1**–**d4** Schematic representation of the four stages of goblet formation, roughly corresponding to (**c1**–**c4**), with the arrows indicating the approximate direction of melt flow (see also simulation in Fig. 6).

However, the biggest surprise of the current study is discovered in the part-crystallized PLA nanocomposite. Figure 5(a1, a2) are *z-*slices at the bottom and the top surface of a film of PLA with labeled 200 nm NPs. The top surface is almost an exact copy of the bottom one, with even the smallest spherulites nearly exactly above each other. Moreover, the mirrored spherulites have almost exactly the same diameter, meaning that they nucleated on opposite surfaces within seconds of each other. How did they communicate?

Observing the 3D morphology in Fig. 5b, revealed by surface rendering, brings us closer to the answer. The picture shows what appears like a treasure find of ancient bowls, vases, and goblets. The objects have C_∞_ symmetry and most have a widened circular base at the bottom and the top; these are believed to be half-spherulites nucleated on opposite surfaces. In panels c1–c4, we present our explanation of the formation of a “goblet,” by showing individual pieces of “crockery” each at a different stage of development. The corresponding schematics are shown in panel d. The formation of a half-spherulite at the top surface (c1, d1) creates negative pressure around it as well as at the surface opposite, where the supply of replacement polymer by diffusion is most limited. This is where nucleation of the second half-spherulite is stress induced (c2, d2). As both half-spherulites grow toward each other a connecting stress field and elongational flow develop^37^ and trigger nucleation and growth of what appears to be shish-kebabs^38^; these initiate the formation of the “stand” of the goblet (c3, d3). In fact, we were fortunate to have captured the beginning of fibrous shish-kebab formation—see inset in Fig. 5(c3). Thus a goblet is formed (Fig. 5(c4, d4)). Had crystallization continued isothermally, it would have ended as a polyhedral column, but its high-crystallinity inner core would have remained goblet shaped, as suggested by the white-line-delineated area in Fig. 3(a3) right.

Other objects in Fig. 5b, with bulging centers, are thought to have nucleated within the film interior but as the initial spherulite grew the negative pressure built up at both nearby surfaces; this had induced additional half-spherulite nucleation, their growth and eventual merger into a column-like object—see several examples in Figs. 3(a4) and 5b. It is likely that most of them include shish-kebab segments, which continued growing laterally to form a “cylindrite” ^39^.

**Fig. 6:**
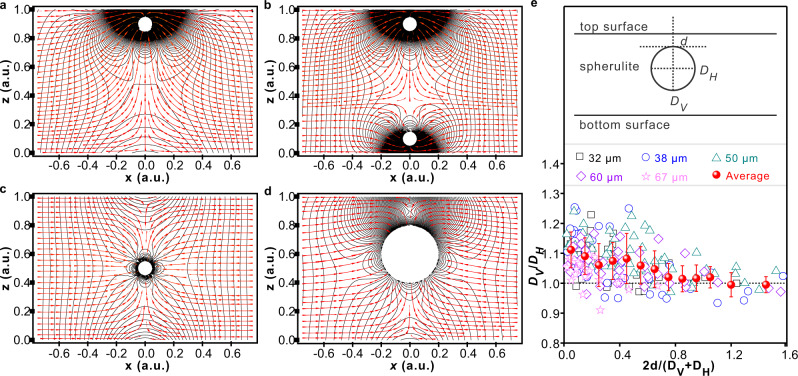
2D numerical simulation of flow and pressure around growing spherulites and the resulting spherulite elongation. **a**–**d** In the 6 × 1 2D simulation box each spherulite is represented by a circular boundary with a constant inflow rate, located at different positions between the top and bottom boundaries, representing the glass plates. Only the central portion of the box is shown. Red arrows = flow lines, black lines = isobars. **a**, One spherulite growing close to the top surface. **b** Two spherulites growing simultaneously at opposite (top and bottom) surfaces. To represent a situation where the top spherulite nucleated earlier and is larger, hence consuming more molten polymer, its inflow rate is three times that of the bottom spherulite. **c** One spherulite grows in the middle of the cell. **d** One spherulite approaches the top surface. The negative pressure between surfaces of the substrate and the spherulite increases as their distance *d* decreases; this is particularly clear above the spherulite in (**d**). **e** Aspect ratio of spherulite of dye-labeled neat PLA *vs*. distance from the nearest glass surface for different sample thicknesses; empty symbols = experimental, red balloons = average. As indicated by the inset, *D*_*V*_ and *D*_*H*_ are diameters along *z* and in *xy* plane, respectively. Error bars correspond to standard deviation.

Numerical simulations of stress distribution and fluid flow in a layer with a fluid sink close to one surface, and two sinks at opposing surfaces, are shown in Fig. 6(a, b), corroborating the above interpretation of the cause of the intriguing observed morphologies. The situation where a spherulite nucleates in the sheet interior is also modeled. The pressure distribution and flow lines shown in Fig. 6c confirm again the negative pressure peaks at both surfaces nearest to the growing spherulite. As the spherulite approaches the surface, the negative pressure increases greatly (Fig. 6d). This explains the appearance of additional surface half-spherulites and their merger with the initial spherulite and the formation of “vases” with a “neck” and columns in Figs. 2(c, d), 3(a3, a4) and 5b.

To our knowledge, this is the first observation of shish-kebab formation in the quiescent melt. Shish-kebabs, or “row-nucleated structures,” normally form in flowing melt or solution, or under externally applied stress, particularly if the polymer has cross-links^38,40^. The stress causing shish-kebab nucleation in the present case is self-generated by spherulite crystallization. The fact that these unusual morphologies are most pronounced in polymers with added NPs can be attributed to the restrictive effect of silica NPs on polymer chain mobility^41–43^. That same effect also causes increased cavitation in NP-containing polymers^16^. The nanoparticles can be regarded here as acting like physical cross-links. In the current experiments, NP concentration is low, while in most practical applications their concentration is higher, thus the effects revealed here are expected to be even more pronounced. However, even without NPs there is the acceleration of spherulite growth when approaching substrate surface, seen both in pure iPP (Fig. 2c) and in PLA (Fig. 2d). Figure 6e clearly shows that the asphericity of spherulites increases with proximity to the substrate even in neat PLA and, to a lesser extent, also in iPP (Supplementary Fig. S8). A PLA spherulite may have a diameter measured vertically up to 25% larger than horizontally. This suggests that the negative pressure^28^ affecting morphology and spherulite growth kinetics is a universal phenomenon in semicrystalline polymers, and in their nanocomposites in particular.

The higher asphericity of PLA spherulites compared to that of iPP can be attributed to lower mobility of PLA in the 120–130 °C temperature range—cf. glass transition temperatures of ∼60 °C for PLA and −10−0 °C for iPP. The slower chain diffusion in PLA will cause the buildup of higher negative pressure. Additional restriction to diffusion in PLA nanocomposite will arise from the interaction of polar PLA with the polar silica, a stronger attraction than that between the silica and the nonpolar iPP. This would explain why the unusual morphologies in Fig. 5 are seen in PLA nanocomposite, and why nonspherical spherulites are mostly observed in PLA and less so in iPP. Effects on 3D morphology of other factors, such as molecular weight and the nature of the polymer, as well as the type, concentration, size, and coating of the NPS, are all subjects of current and future investigations.

### Conclusions and outlook

We have shown how morphology of semicrystalline polymers and their nanocomposites can be visualized in 3D on the critical 10–1000 µm scale. The first 3D images of growing and fully grown spherulites have revealed that they are not always spherical, that in nanocomposites their nucleation can be copied across from one surface to another through growth-induced negative pressure, and that this can induce shish-kebabs formation in the static melt. All this results in morphologies that escaped previous notice due to a lack of 3D imaging. The technique is expected to be applied to other systems, revealing further unknown morphological features of polymers and nanocomposites.

## Methods

### Materials

iPP (*M*_w_ = 2.5 × 10^5^ g/mol and *M*_n_ = 6.7 × 10^4^ g/mol), Nile Red (C_20_H_18_N_2_O_2_), anhydrous p-*xy*lene 99%, and dioxane were purchased from Sigma-Aldrich. PLLA 4032D containing around 2% D-lactide (*M*_w_ = 2.23 × 10^5^ g/mol and *M*_n_ = 1.06 × 10^5^ g/mol) was obtained from NatureWorks (USA). The syntheses of the Nile Red derivative 9-diethylamino-2-(triethoxysilyl-3-propyloxy)-5H-benzo[α]phenoxazin-5-one and SiO_2_ NPs of 200 nm were carried out according to refs. ^44,45^, respectively. Their hybridization is described in ref. ^16^.

### Sample preparation

Dye- and NP-doped polymers were prepared by freeze-drying mixed solutions in order to obtain a uniform mixture of NR and the polymers. For iPP + NR sample, iPP pellets and NR powder (2000:1 wt ratio) were first dissolved in p-xylene (130 °C, mild stirring). The solution was frozen by quenching in liquid nitrogen, after which the solvent was sublimed off under vacuum at 0 °C. For PLA + NR sample, the solvent was dioxane and the solution mixing temperature was 50 °C. The final concentration of NR in both the iPP and PLA was 0.05 wt%. Supplementary figure S1 illustrates the uniformity of the polymer-dye blends.

For polymer-nanoparticle blends NPs were first dispersed in methanol (sonication, 60 min), then the NP suspension was mixed with polymer solution followed by freeze-drying. The solvents and dissolution temperatures were the same as above. The concentration of 200 nm NPs in both the iPP and PLA was 1.3 wt%.

For crystallization of dye- and NP-doped polymers the samples were placed between a glass slide and a coverslip, annealed at 210 °C for 10 min to erase thermal history. They were then cooled to *T*_c_ at the highest cooling rate allowed by the hot stage (ca 30 °C/min) and maintained at *T*_c_ for a set period. Afterwards, the samples were quenched in ice water.

Dye-labeling by immersion of neat iPP sheets crystallized at 130 °C for 12 min was carried out by placing the sheet in a flask with NR/p-xylene solution (6 mg/L). The temperature was set at 25 °C for the penetration of NR into iPP film, as this temperature left the crystalline regions unaffected^46,47^.

### Microscopy

Two-photon confocal fluorescence microscopy was done on an Upright Zeiss LSM 510 META confocal microscope equipped with a Chameleon Ti-Sapphire femtosecond pulsed laser with a wavelength of 1000 nm, an oil-immersion 40 × /1.30 objective, and a HFT KP650 dichroic mirror accepting laser wavelength >650 nm and allowing through fluorescent emission <650 nm. *xy* plane slices were captured by line-by-line scanning and moved along *z-*axis direction 1 μm steps. The 3D rendering methods are described in figure captions.

Scanning electron microscopy was performed on a field-emission instrument (Inspect F, FEI, USA), operating at 5 kV. Small pieces of ~200 μm thick neat PLA and PLA + NP 200 nm samples were melt crystallized at 130 °C for 10 min and immersed in liquid nitrogen for 1 h, then cryogenically fractured. The smooth fracture surface was sputter-coated with gold.

### Finite element analysis

FEA of negative pressure and melt flow caused by spherulite growth is carried out using Mathematica, by solving the Navier-Stokes equations in a 2D box of size 6 × 1. The growing spherulites are represented by circular boundaries at coordinates (0,z), with *z* = 0.1 (close to the bottom surface), 0.5 (middle of the box), 0.6 (slightly above the middle) or 0.9 (close to the top surface) respectively, with a radius of 0.05 or 0.2 and a constant outflow rate at the boundary. Two counterbalancing inflowing boundaries are placed close to the two side edges of the box, at coordinates (±2.5, 0.5). The flow rates are set to zero at the four straight boundaries of the box. The equations are solved numerically in iterations until the estimated normalized error is smaller than 10^−5^ for the dataset.

## Supplementary information

Supplementary Information

Description of Additional Supplementary Files

Supplementary Movie 1

Supplementary Movie 2

Supplementary Movie 3

Supplementary Movie 4

Supplementary Movie 5

Supplementary Movie 6

## Data Availability

The raw images (.lsm files) generated in this study are available for download at the figshare database, 10.6084/m9.figshare.14910291
