# Supplementary material for: Bowls, vases and goblets—the microcrockery of polymer and nanocomposite morphology revealed by two-photon optical tomography
Source: Nat Commun. 2021 Aug 20;12:5054. doi: 10.1038/s41467-021-25297-w (PMC8379155; doi:10.1038/s41467-021-25297-w)
Supplement: Supplementary file 1 — Supplementary Information [file 41467_2021_25297_MOESM1_ESM.pdf]

Supporting Information for:

Bowls, Vases and Goblets – The Microcrockery of Polymer and Nanocomposite Morphology Revealed by Two-Photon Optical Tomography

Shu-Gui Yang[1,2], Zhen-Zhen Wei[2,3], Liliana Cseh[4], Pantea Kazemi[2], Xiang-bing Zeng[2], Hui-Jie Xie[5], Hina Saba[5], Goran Ungar[1,2*]

1. State Key Laboratory for Mechanical Behaviour of Materials, Shaanxi International Research Centre for Soft Matter, Xi'an Jiaotong University, Xi'an, 710049, P. R. China. Email: g.ungar@xjtu.edu.cn;

2. Department of Materials Science and Engineering, University of Sheffield, Sheffield S1 3JD, UK. g.ungar@sheffield.ac.uk

3. College of Textile and Clothing Engineering, National Engineering Laboratory for Modern Silk, Soochow University, Suzhou 215123, China

4. Romanian Academy, Coriolan Dragulescu Institute of Chemistry, Timisoara 300223, Romania

5. Department of Physics, Zhejiang Sci-Tech University, 310018, Hangzhou, China

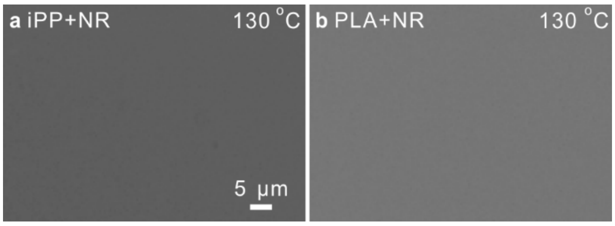

Fig. S1. a, iPP+NR and **b**, PLA+NR at 130 °C before spherulite formation. As can be seen, fluorescence is completely uniform, showing no aggregation of the Nile Red dye.

Figure S2a shows a transmission electron microscope (TEM) image of the 500 nm diameter silica nanoparticles used. **Figure S2b** shows that a NR molecule contained a siloxane head group is chemically bound to the NP. **Figure S2c** is the histogram of NP size measured from TEM images. It shows that the NPs are highly monodisperse.

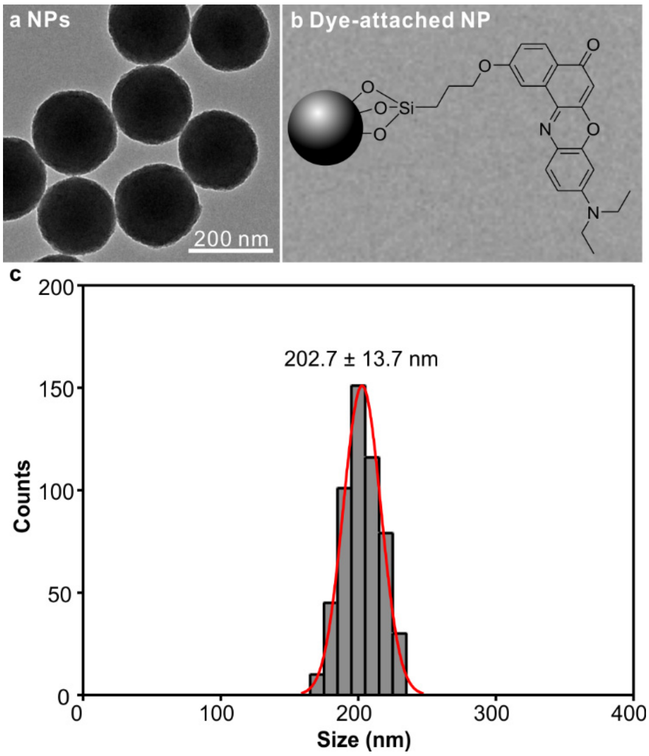

Fig. S2 a, TEM image of SiO_2 NPs of 200 nm diameter. **b**, Siloxane-modified NR molecule chemically bonded to a SiO_2 NP. **c**, Size distribution of the NPs.

To find the suitable immersion time for labelling iPP sample with NR, different immersion times were explored. **Figures S3a, S3b** and **S3c, S3d** show the examples of under- (14 and 18 hours) and over-immersion (40 and 65 hours).

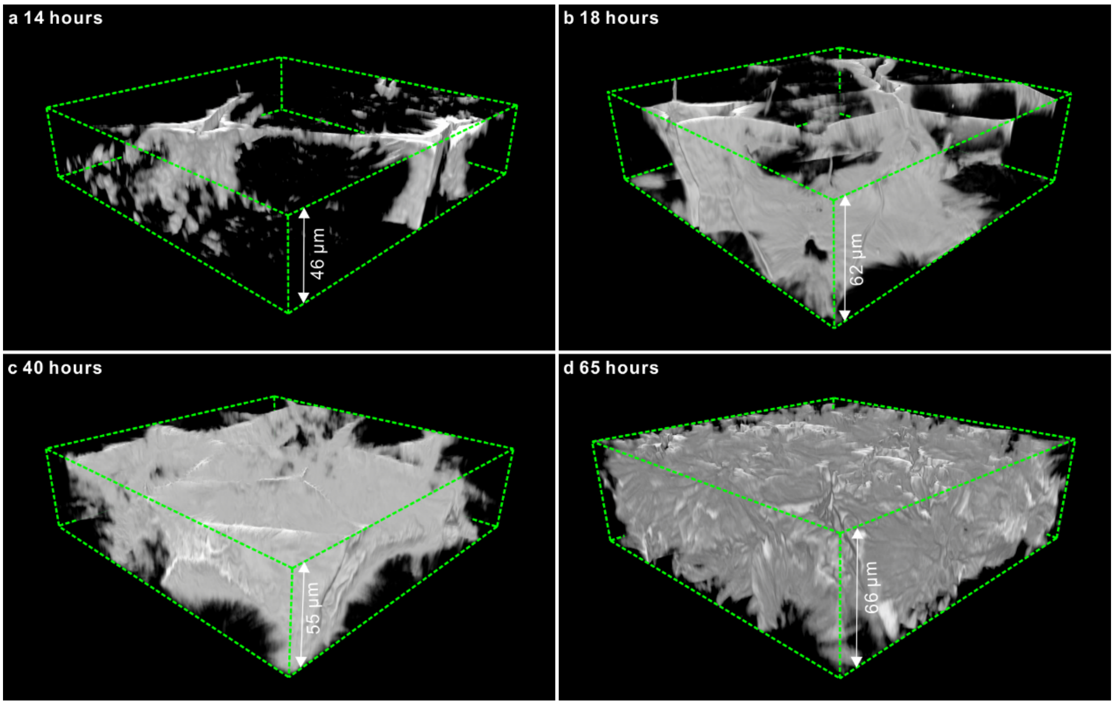

Fig. S3 "Porous" 3D rendering of iPP sample labelled by infiltration of the NR solution, **a** (14 hours) and **b** (18 hours) under-immersion, **c** (40 hours) and **d** (65 hours) over-immersion.

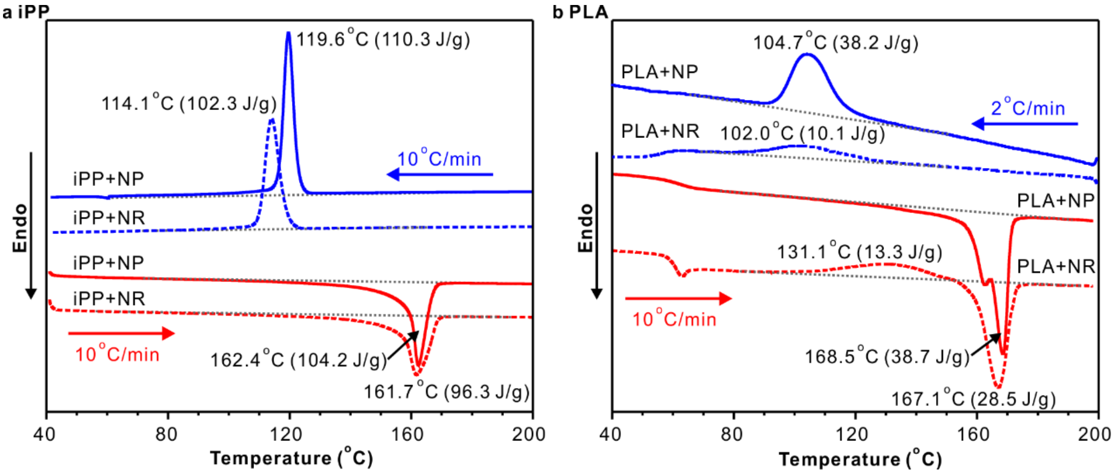

Fig. S4. Heating and cooling DSC scans of iPP (a) and PLA (b) blends with Nile Red and with 200 nm nanoparticles. Full lines: blends with NPs, dashed line: blends with dye only.

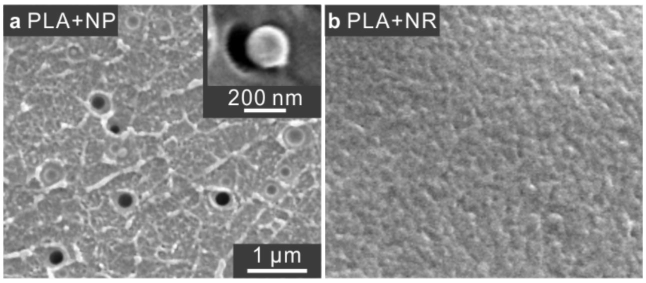

Fig. S5 SEM images of **a**, PLA+NP and **b**, PLA+NR quenched to room temperature from melt. The holes are the result of individual NPs having been pulled out. The inset in (**a**) shows a NP partially pulled out from the bulk.

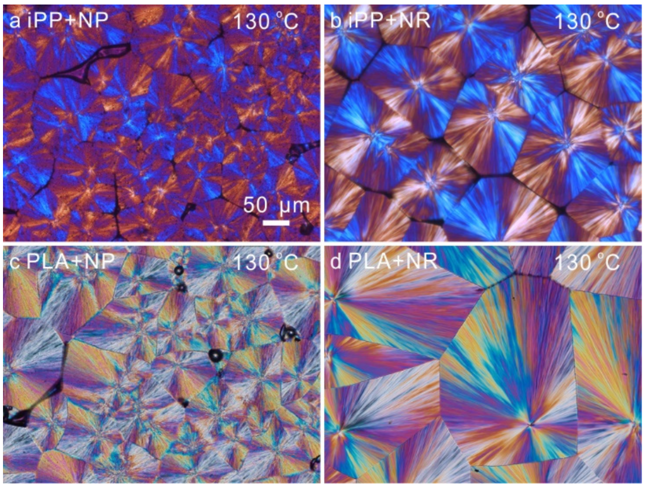

Fig. S6 POM images of **a**, iPP+NP, **b**, iPP+NR, **c**, PLA+NP, **d**, PLA+NR, all crystallized at 130 °C. The addition of NP increases spherulite nucleation both in iPP and in PLA.

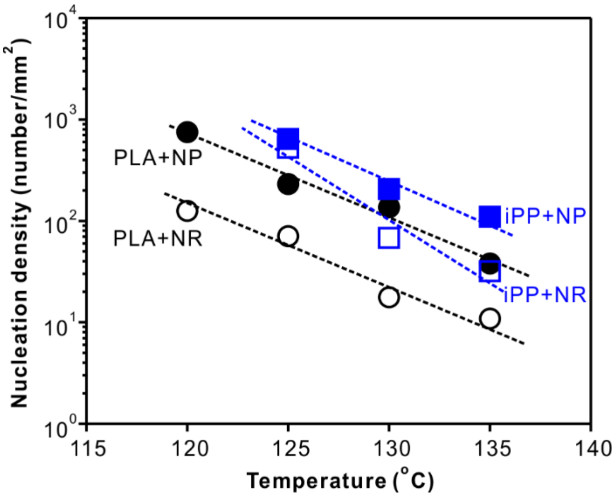

Fig. S7 Nucleation densities of iPP+NP, iPP+NR, PLA+NP, PLA+NR as a function of crystallization temperature. As can be seen, the effect of NPs on nucleation is considerably more significant in PLA compared to that in iPP.

Figure S8 shows that, as in PLA, the aspect ratio of iPP spherulites also increases with the proximity of the substrate, but to a lesser extent.

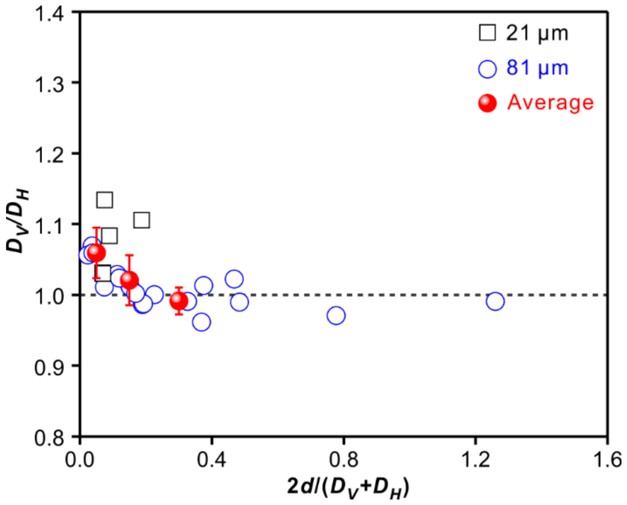

Fig. S8 Aspect ratio of growt-arrested iPP spherulites as a function of relative distance from the glass surface (*cf.* Fig 6e).
