# Supplementary material for: Bowls, vases and goblets—the microcrockery of polymer and nanocomposite morphology revealed by two-photon optical tomography
Source: Nat Commun. 2021 Aug 20;12:5054. doi: 10.1038/s41467-021-25297-w (PMC8379155; doi:10.1038/s41467-021-25297-w)
Supplement: Supplementary file 2 — Description of Additional Supplementary Files [file 41467_2021_25297_MOESM2_ESM.pdf]

Description of Additional Supplementary Files

File Name: Supplementary Movie 1
Description: Fully crystallized iPP+NR. - Isotactic polypropylene (iPP) labelled with Nile Red (NR), fully cryst allized, internal structure of a block using confocal microscopy. Spherulite centres are dark-blue/black, spherulite boundaries are yellow. See Fig. 1c.

File Name: Supplementary Movie 2
Description:
PP+NR - Dye labelling by infiltration. - Neat iPP was crystallized at 130 C and subsequently immersed in NR/p-xylene solution; "porous" 3D rendering, highlighting the strongly fluorescent regions of the dye that preferen tially stained the spherulite boundaries. See Fig. 1d1.

File Name: Supplementary Movie 3
Description: Part-crystallized iPP+NR - iPP +Nile Red, isothermally crystallized at Tc = 135 C for 12 min, then quenched in ice-water, showing spherulites whose growth had been arrested. See Fig. 2a2.

File Name: Supplementary Movie 4
Description: Part-crystallized PLA+NR - Same as Video 3 but with poly(lactic acid) (PLA) with NR dye. Tc = 130 C. See Fig. 2b2.

File Name: Supplementary Movie 5
Description: Fully crystallized PLA+NP - 3D morphology of fully crystallized PLA with NR-labelled 200 nm silica nanoparticles (NP). "Porous" 3D rendering , highlighting the strongly fluorescent regions of the nanoparticles segregated at spherulite boundaries during crystallization. See Fig. 3a2.

File Name: Supplementary Movie 6
Description: Part-crystallized PLA+NP - 34 micron thick film of PLA containing 1.3 wt% of 200 nm NR-labelled silica NPs, crystallized at Tc = 130 C for 10 min; spherulite growth was then arrested by quenching in ic e water. Surface rendering of arrested spherulite growth. The morphology resembles a treasure of ancient crockery.
